# PLA2G4A and ACHE modulate lipid profiles via glycerophospholipid metabolism in platinum-resistant gastric cancer

**DOI:** 10.1186/s12967-024-05055-4

**Published:** 2024-03-07

**Authors:** Menglin Chen, Cancan Zhang, Huaizhi Li, Shanshan Zheng, Yaqi Li, Mengyun Yuan, Yuxuan Chen, Jian Wu, Qingmin Sun

**Affiliations:** 1 Jiangsu Province Key Laboratory of Tumor Systems Biology and Chinese Medicine, Jiangsu Province Hospital of Chinese Medicine, Affiliated Hospital of Nanjing University of Chinese Medicine, 155 Hanzhong Road, Nanjing, 210029 Jiangsu China; 2 No.1 Clinical Medical College, Nanjing University of Chinese Medicine, Nanjing, 210023 Jiangsu China

**Keywords:** Lipidomic, Gastric cancer, Platinum chemotherapy, Biomarkers, Resistance

## Abstract

**Background:**

Bioactive lipids involved in the progression of various diseases. Nevertheless, there is still a lack of biomarkers and relative regulatory targets. The lipidomic analysis of the samples from platinum-resistant in gastric cancer patients is expected to help us further improve our understanding of it.

**Methods:**

We employed LC–MS based untargeted lipidomic analysis to search for potential candidate biomarkers for platinum resistance in GC patients. Partial least squares discriminant analysis (PLS-DA) and variable importance in projection (VIP) analysis were used to identify differential lipids. The possible molecular mechanisms and targets were obtained by metabolite set enrichment analysis and potential gene network screened. Finally, verified them by immunohistochemical of a tissue microarray.

**Results:**

There were 71 differential lipid metabolites identified in GC samples between the chemotherapy-sensitivity group and the chemotherapy resistance group. According to Foldchange (FC) value, VIP value, P values (FC > 2, VIP > 1.5, p < 0.05), a total of 15 potential biomarkers were obtained, including MGDG(43:11)-H, Cer(d18:1/24:0) + HCOO, PI(18:0/18:1)-H, PE(16:1/18:1)-H, PE(36:2) + H, PE(34:2p)-H, Cer(d18:1 + hO/24:0) + HCOO, Cer(d18:1/23:0) + HCOO, PC(34:2e) + H, SM(d34:0) + H, LPC(18:2) + HCOO, PI(18:1/22:5)-H, PG(18:1/18:1)-H, Cer(d18:1/24:0) + H and PC(35:2) + H. Furthermore, we obtained five potential key targets (PLA2G4A, PLA2G3, DGKA, ACHE, and CHKA), and a metabolite-reaction-enzyme-gene interaction network was built to reveal the biological process of how they could disorder the endogenous lipid profile of platinum resistance in GC patients through the glycerophospholipid metabolism pathway. Finally, we further identified PLA2G4A and ACHE as core targets of the process by correlation analysis and tissue microarray immunohistochemical verification.

**Conclusion:**

PLA2G4A and ACHE regulated endogenous lipid profile in the platinum resistance in GC patients through the glycerophospholipid metabolism pathway. The screening of lipid biomarkers will facilitate earlier precision medicine interventions for chemotherapy-resistant gastric cancer. The development of therapies targeting PLA2G4A and ACHE could enhance platinum chemotherapy effectiveness.

## Introduction

Gastric cancer (GC) represents one of the most lethal malignancies globally, due to a lack of early symptoms, many patients are diagnosed with locally advanced disease, which seriously impairs patient survival [1]. The median survival duration is between 12 and 15 months [2, 3]. Despite progress in initial detection and clinical intervention, the prognosis for advanced GC patients remains unsatisfactory [4]. The treatment for advanced GC typically involves sequential chemotherapy regimens, with platinum chemotherapy agents often being widely used as first-line treatment options [1]. Nevertheless, chemoresistance presents a significant hurdle for the optimal prognosis of GC patients due to the deficiency of effective biomarkers [5]. Therefore, the discovery of biomarkers that could be used to detect chemotherapy resistance in GC would greatly assist in clinical diagnosis and patient treatment.

Supported by the close connection between altered lipid metabolism and the pathogenic process, specific lipid profiles are emerging as unique disease biomarkers, with diagnostic, prognostic and predictive potential [6]. High throughput lipidomics and computational biology techniques serve as potent tools in the personalized examination of tumor lipid signatures and unveiling of cancer-associated biomarkers [7]. Sophisticated computational methods provide significant advantages in interpreting large-scale data, leading to a deeper understanding of metabolic processes and their underlying mechanisms [8]. It was found that phosphatidylethanolamine (PE) (36:3), PE (36:2), phosphatidylcholines (PC) (32:0), and sphingomyelin (SM) (d18:0/18:1(9Z)) were more abundant in patients with early gastric cancer than in a study investigating early markers of GC [9]. However, alterations in lipid metabolites linked to platinum-resistant GC and the respective underlying gene regulatory mechanisms remain unreported.

The increasing research indicated that metabolic adaptations may also bolster resistance to both chemotherapy and targeted agents. The reprogramming of lipid metabolism, a process integral to energy utilization and cellular signaling, promotes cell survival and facilitates the onset of multidrug resistance (MDR) in cancer cells [10–14]. Notably, lipids, including phospholipids and cholesterol, compose the plasma membrane and partake in the resistance mechanism by modulating the activity of ATP-binding cassette (ABC) multidrug efflux transporters [7]. PLA2G4A is a member of the cytosolic phospholipase A2 family, which is involved in the release of arachidonic acid from membrane phospholipids, and it subsequently participates in the biosynthesis of prostaglandins and leukotrienes [15]. Some evidence pointed to high PLA2G4A expression in gastric cancer, and inhibition of PLA2G4A has been proposed as a therapeutic strategy to counteract chemo-resistance in this malignancy [16]. At the same time, ACHE is an enzyme that catalyzes the breakdown of acetylcholine and terminates synaptic transmission, mainly associated with neurological function [17]. Inhibiting ACHE enzyme activity could decrease the proliferation and metastasis of gastric cancer cells [18]. However, the mechanism by which PLA2G4A and ACHE on platinum resistance in gastric cancer has not been explored.

To preliminarily explore whether PLA2G4A and ACHE disrupt platinum-resistant lipid disorders in gastric cancer through glycerophospholipid metabolism, we undertook an exhaustive analysis of the lipid profile in 21 GC patients with platinum chemotherapy resistance (CR) and 8 with platinum chemotherapy sensitivity (CS) using LC–MS-based untargeted lipidomics. We identified 15 lipid metabolites as potential biomarkers. Simultaneously, PLA2G4A and ACHE regulatory genes were determined via KEGG functional enrichment, gene screening, correlation analysis and immunohistochemistry of tissue microarray. Our study also provides a theoretical basis for the development of future drugs to alleviate platinum resistance.

## Materials and methods

### Chemicals and reagents

MS-grade methanol (047192), MS-grade acetonitrile (51101), HPLC-grade 2-propanol (022906) were purchased from ThermoFisher (USA). HPLC-grade formic acid (5438040250) and HPLC-grade ammonium formate (714690-4X4L) were purchased from Sigma (Germany).

#### Patients and sample collection

A total of 29 GC tissues (21 cases of oxaliplatin sensitivity and 8 cases of oxaliplatin resistance) were obtained from patients who underwent postoperative adjuvant chemotherapy with oxaliplatin‐based regimens (FOLFOX or XELOX). Follow‐up was conducted regularly via telephone or mail. Patients with local relapse were categorized as oxaliplatin‐resistant. Detailed clinicopathological characteristics are listed in Table 1. Histopathology results for all cancer patients were confirmed by surgical resection of the tumors, while clinical characteristics and tumor stages were assessed based on biopsy results. Each patient’s age and clinical manifestations were statistically analyzed, and informed consent was obtained. All samples were collected in accordance with ethical guidelines, and written informed consent was obtained. All clinical experiments were approved by the Independent Ethics Committee of the Affiliated Hospital of Nanjing University of Chinese Medicine, Number: 2017NL-092–02. All patients were approached based on approved ethical guidelines, and patients who agreed to participate in this study signed consent forms before being included in the study. We also confirmed that all methods were performed in accordance with the relevant guidelines and regulations. All studies were conducted in accordance with the principles of the Declaration of Helsinki.

**Table 1:** Information of platinum-chemotherapy gastric cancer patients

Characteristics		Chemotherapy sensitivity n = 21	Chemotherapy resistance n = 8
Gender			
Female	9	8	1
Male	20	13	7
Age			
< 60	12	10	2
≥ 60	17	11	6
Lauren classification			
Intestinal	5	4	1
Diffuse and mixed	23	16	7
Not stated	1	1	0
Depth of tumor invasion			
Localized in subserosa	7	5	2
Beyond subserosa	18	12	6
Not stated	4	4	0
Lymph node metastasis			
N0	7	5	2
N1-3	18	12	6
Not stated	4	3	0
pTNM stage			
I/II	10	8	2
III/IV	15	9	6
Not stated	4	4	0
Vascular invasion			
Yes	18	10	8
No	12	11	1

#### Sample preparation and lipid extraction

Lipids were extracted according to the MTBE method. Briefly, samples were homogenized in 200 µL of water and 240 µL of methanol. Then 800 µL of MTBE was added and the mixture was sonicated 20 min at 4 ℃ followed by sitting still for 30 min at room temperature. The solution was centrifuged at 14000 g for 15 min at 10 ℃ and the upper organic solvent layer was obtained and dried under nitrogen.

#### LC–MS/MS method for lipid analysis

Reverse-phase chromatography was employed for LC separation using CSH C18 column (1.7 µm, 2.1 mm × 100 mm, Waters). The lipid extracts were redissolved in 200 µL 90% isopropanol/ acetonitrile, centrifuged at 14000 g for 15 min, finally, 3 µL of the sample was injected. Solvent A was acetonitrile–water (6:4, v/v) with 0.1% formic acid and 0.1 mM ammonium formate and solvent B was acetonitrile–isopropanol (1:9, v/v) with 0.1% formic acid and 0.1 mM ammonium formate. The initial mobile phase was 30% solvent B at a flow rate of 300 μL/min. It was held for 2 min, and then was linearly increased to 100% solvent B over 23 min, followed by equilibration at 5% solvent B for 10 min. The data were processed using Simca P14.1 software for multivariate statistical analysis, including principal component analysis (PCA) and orthogonal partial least squares discriminant analysis (OPLS-DA). The OPLS-DA models were validated with a permutation testing and cross-validation. Variable importance projection values (VIP > 1.5) and t-test p-values (p < 0.05) were considered as differential lipids. Moreover, thresholds are established for lipids identified using LipidSearch software to reduce unreliable results, which improves the accuracy. Single-point internal standard calibrations were used to estimate absolute concentrations for lipids identified by accurate mass, MS/MS spectral match, and retention times.

#### KEGG and receiver operating characteristic (ROC) curve analyses

MetaboAnalyst 5.0 (https://www.metaboanalyst.ca/) was used for the pathway enrichment analysis and ROC curve analyses of the potential biomarkers. The area under the curve (AUC) of the ROC curve was calculated to quantify the accuracy.

#### Network analyzes

A total of 2327 disease targets were identified using “Platinum resistance” as the keyword in the GeneCards database. A total of 99 Glycerophospholipid metabolism-related genes were extracted from the KEGG database and 5 targets related to platinum resistance were finally obtained. The potential lipid biomarkers were imported into Metscape 3.7.2 to generate a metabolite-gene network. The protein–protein interaction (PPI) network was constructed by inputting the predicted targets into the STRING database (https://string-db.org/). Based on the PPI results, we focused on their linked targets to discover the underlying mechanisms. Consequently, the network elucidating the relationships between metabolites and corresponding genes was established using Metscape. Through the analysis of this network, key targets within the interaction network were selected for further analysis.

#### Immunohistochemical (IHC) analysis

Immunohistochemical (IHC) analysis was carried out to assess GC tumor tissues. We collected 99 pairs GC samples from patients who were treated by surgical removal for tissue microarray (TMA). TMA included 12 pairs of patients in this study, which 7 pairs belongs to chemotherapy sensitive group and 5 pairs belongs to chemotherapy resistant group. All clinical samples were collected at Affiliated Hospital of Nanjing University of Chinese Medicine and verified by histological and pathological examinations. All participating patients had provided written informed consent. Immunohistochemical staining by using the following primary antibodies at the indicated dilution: PLA2G4A 1:100 (Zenbio Cat# 822144), DGKA 1:100 (Zenbio Cat# R26679) and ACHE (Zenbio Cat# 160022)**.**

#### Statistical analysis

Data were presented as mean ± standard deviation. All the performed statistical analyses are described in each figure legend. Statistical p-values were obtained by application of the appropriate statistical tests using the GraphPad Prism 8. For all tests, p < 0.05 was considered significant (*p < 0.05, **p < 0.01).

## Results

### Intrinsic lipid metabolic exerted variations in the platinum resistance GC patients

**Fig. 1:**
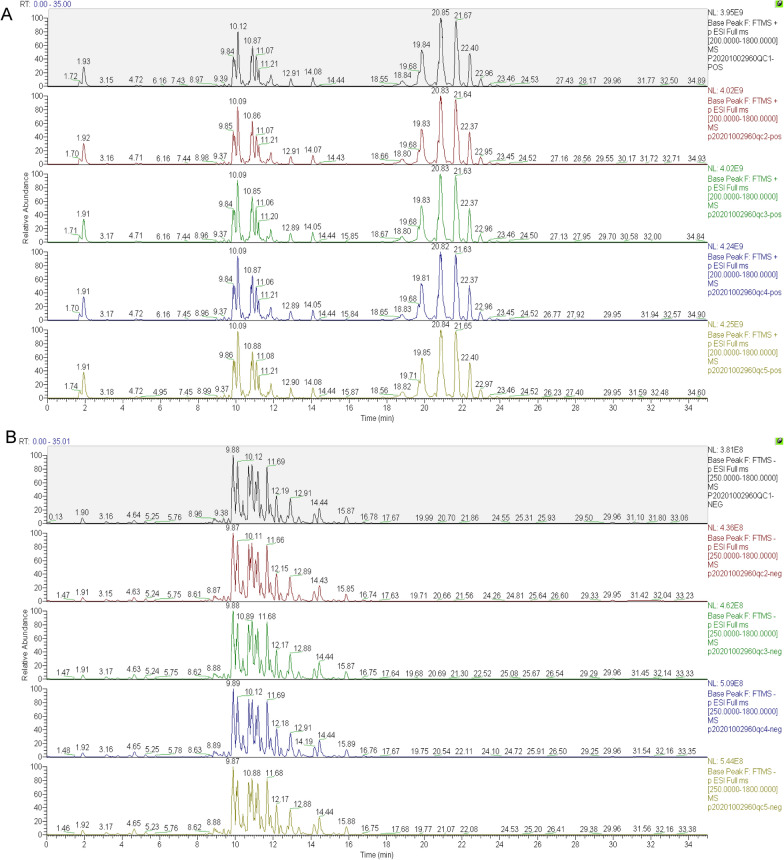
Comparison of Base Peak chromatogram (BPC) of QC samples. **A** Sample positive ion pattern BPC overlap map **B** Sample negative ion pattern BPC overlap map

The base peak chromatogram (BPC) diagram of the quality control (QC) sample displayed spectral overlap, as shown in the Fig. 1A and B. The experimental results showed that the chromatographic peak response intensity and retention time of QC samples basically overlap, indicating good experimental repeatability.

**Fig. 2:**
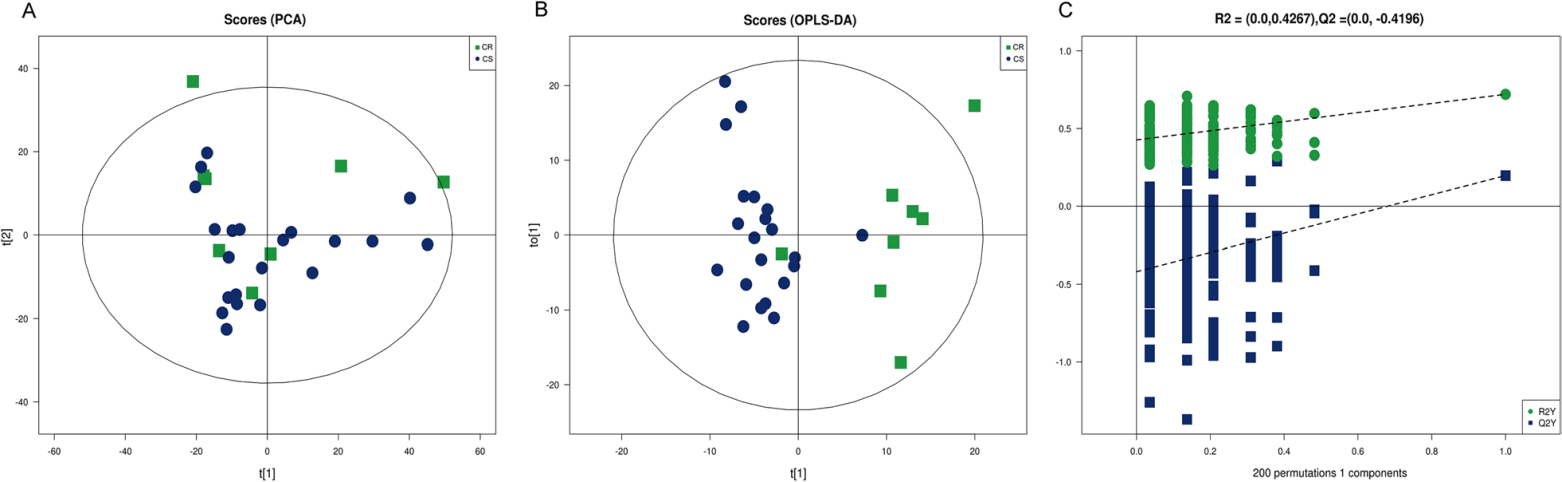
Multivariate analysis of untargeted lipidomic research. **A** PCA score plots of metabolic profiles in chemotherapy sensitivity and chemotherapy resistance groups. **B** Score plots of OPLS-DA of the normalized LC–MS data from the chemotherapy sensitivity and chemotherapy resistance groups. **C** Corresponding permutation analyses for the statistical validation of the OPLS-DA models

Additionally, an unsupervised principal component analysis (PCA) model was employed to examine the dispersion pattern. The PCA score plots showed that QC samples clustered tightly under both the positive and negative ion models, indicating that the instrument functions quite well. The PCA score plots (Fig. 2A) showed a clear trend of separation of the model group and control group, indicating significant differences in the endogenous lipid profiles of GC patients with platinum chemotherapy resistance. Compared with the chemotherapy sensitivity (CS), the chemotherapy resistance (CR) group was significantly separated, indicating that there were significant differences in endogenous lipids in patients with platinum chemotherapy resistance in GC. The cumulative fitness (R2 value) of the PCA model was 0.597, indicating a suitable model fit. Moreover, The OPLS-DA supervised pattern recognition method was performed further to identify potential biomarkers related to patients with platinum chemotherapy resistance in GC. The OPLS-DA analysis indicated clear separations between the chemotherapy-sensitivity (green dots) and chemotherapy-resistance (blue dots) groups (R2Y = 0.72, Q2 = 0.197, Fig. 2B). The results of the permutation test strongly indicated that the original model was valid (R2 intercept = 0.4267, Q2 intercept = -0.4196, Fig. 2C). These results indicated that our experimental model can be used for lipid profile changes in platinum-resistant GC patients.

### Glycerophospholipid metabolism could affect the endogenous lipids in platinum resistance GC patients

**Fig. 3:**
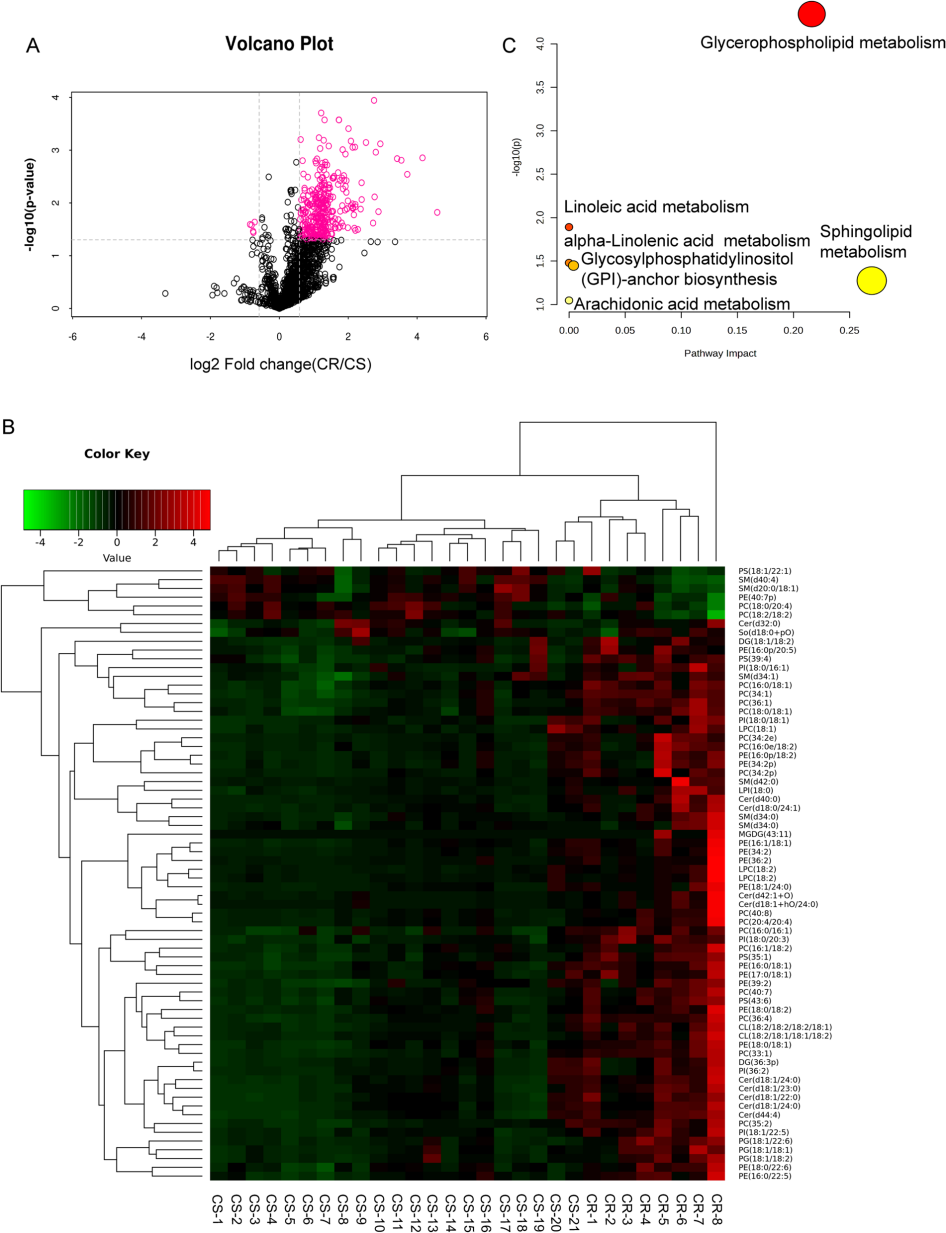
Glycerophospholipid metabolism could affect the endogenous lipids in platin-resistant GC patients. **A** Volcano map showed the differential lipids (VIP > 1.5 and P < 0.05 and fold change > 1.5 or < 0.67). **B** Cluster heat map of differential lipids in chemotherapy sensitivity and chemotherapy resistance group. **C** Lipid metabolic pathway analysis based on significantly differential lipids in chemotherapy sensitivity versus chemotherapy resistance groups

Based on the selection criteria (VIP > 1.5 and P < 0.05 and fold change > 1.5 or < 0.67), 71 differential lipids were obtained. The volcano plot could directly indicate the overall differential expression ratio of lipid molecules in the comparison group. The volcano plot comparing chemotherapy-sensitive vs. chemotherapy-resistant data was shown in Fig. 3A. The rose-red areas in the plot highlight the differential lipid molecules identified by univariate statistical analysis. Hierarchical cluster analysis furthermore revealed the variable values. As depicted in Fig. 3B, the plot provided a direct visual representation of the differential lipid changes between the chemotherapy-sensitive and chemotherapy-resistant groups.

Next, to elucidate the potential pathway of these differential lipids in chemotherapy-GC patients, we charted these differential metabolites of lipids into their biochemical pathways using metabolic enrichment and pathway analyses based on the KEGG database and MetaboAnalyst. As shown in Fig. 3C, KEGG pathway enrichment analysis identified five potential pathways including glycerophospholipid metabolism, Linoleic acid metabolism, alpha-linolenic acid metabolism, Glycosylphosphatidylinositol (GPI)-anchor biosynthesis, Sphingolipid metabolism, Arachidonic acid metabolism. Notably, glycerophospholipid metabolism was identified as the most significantly altered pathway. In conclusion, alterations in glycerophospholipid metabolism may affect the endogenous lipids of platinum resistance in GC patients.

### 15 differential lipid metabolites as potential biomarkers in platinum-resistant GC patients

**Fig. 4:**
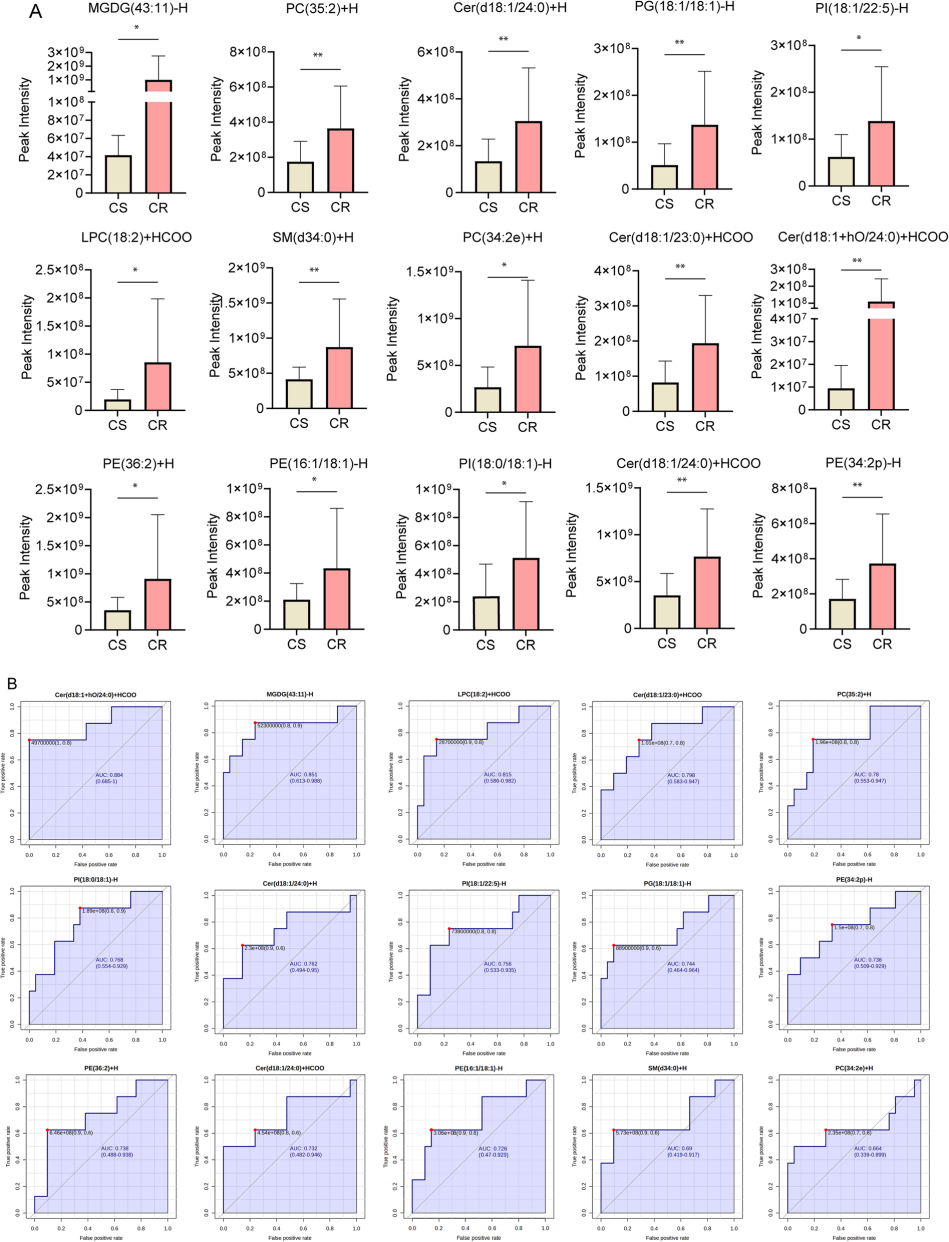
Peak intensity and ROC curves of 15 potential markers. **A** Peak intensity maps of 15 potential markers. **B** ROC curves of 15 potential markers. Arrange from high to low values based on AUC values from MetaboAnalyst

To refine the selection of biomarkers, we increased VIP and FC values to screen |FC|> 2 and VIP > 1.5 as potential biomarkers. A total of 15 potential biomarkers were obtained (Table 2). As shown in Fig. 4A, MGDG(43:11)-H, Cer(d18:1/24:0) + HCOO, PI(18:0/18:1)-H, PE(16:1/18:1)-H, PE(36:2) + H, PE(34:2p)-H, Cer(d18:1 + hO/24:0) + HCOO, Cer(d18:1/23:0) + HCOO, PC(34:2e) + H, SM(d34:0) + H, LPC(18:2) + HCOO, PI(18:1/22:5)-H, PG(18:1/18:1)-H, Cer(d18:1/24:0) + H, PC(35:2) + H were potential biomarkers that match the screening. Meanwhile, they were significantly increased in chemotherapy resistance groups. Subsequently, for more rigorous and accurate identification of potential biomarkers of chemotherapy resistance, we uploaded these 15 biomarkers to MetaboAnalyst and conducted Receiver Operating Characteristic Curve (ROC) curves. A metabolite with an AUC ≥ 0.7 is considered to have moderate or higher diagnostic value. Notably, the AUC for Cer(d18:1 + hO/24:0) + HCOO was 0.887, the highest among our biomarkers. We ranked the AUC area of these candidate markers from highest to lowest (Fig. 4B). Ultimately, it was found that, of the candidate markers, only SM(d34:0) + H and PC(34:2e) + H had a low diagnostic value. These results indicated that the biomarkers we have identified possess diagnostic value and may serve as potential markers for the clinical diagnosis of platinum-resistant GC.

**Table 2:** 15 potential lipid biomarker information

Classification	Class	Name	Foldchange
Monogalactosyldiacylglycerol	MGDG	MGDG(43:11)-H	23.98185741
Ceramide	Cer	Cer(d18:1/24:0) + HCOO	2.171698636
Phosphoinositide	PI	PI(18:0/18:1)-H	2.123430427
Phosphatidylethanolamine	PE	PE(16:1/18:1)-H	2.062432856
Phosphatidylethanolamine	PE	PE(36:2) + H	2.593692118
Phosphatidylethanolamine	PE	PE(34:2p)-H	2.176050791
Ceramide	Cer	Cer(d18:1 + hO/24:0) + HCOO	11.64999933
Ceramide	Cer	Cer(d18:1/23:0) + HCOO	2.362004486
Phosphatidylcholine	PC	PC(34:2e) + H	2.661921223
Sphingomyelin	SM	SM(d34:0) + H	2.110219457
Lysophosphatidylcholine	LPC	LPC(18:2) + HCOO	4.395238596
Phosphoinositide	PI	PI(18:1/22:5)-H	2.22989599
Phosphatidyl glycerol	PG	PG(18:1/18:1)-H	2.678431893
Ceramide	Cer	Cer(d18:1/24:0) + H	2.279011683
Phosphatidylcholine	PC	PC(35:2) + H	2.081638556

### PLA2G4A, PLA2G3, DGKA, ACHE, and CHKA could be potential targets for regulating endogenous lipids in platinum-resistant GC patients

**Fig. 5:**
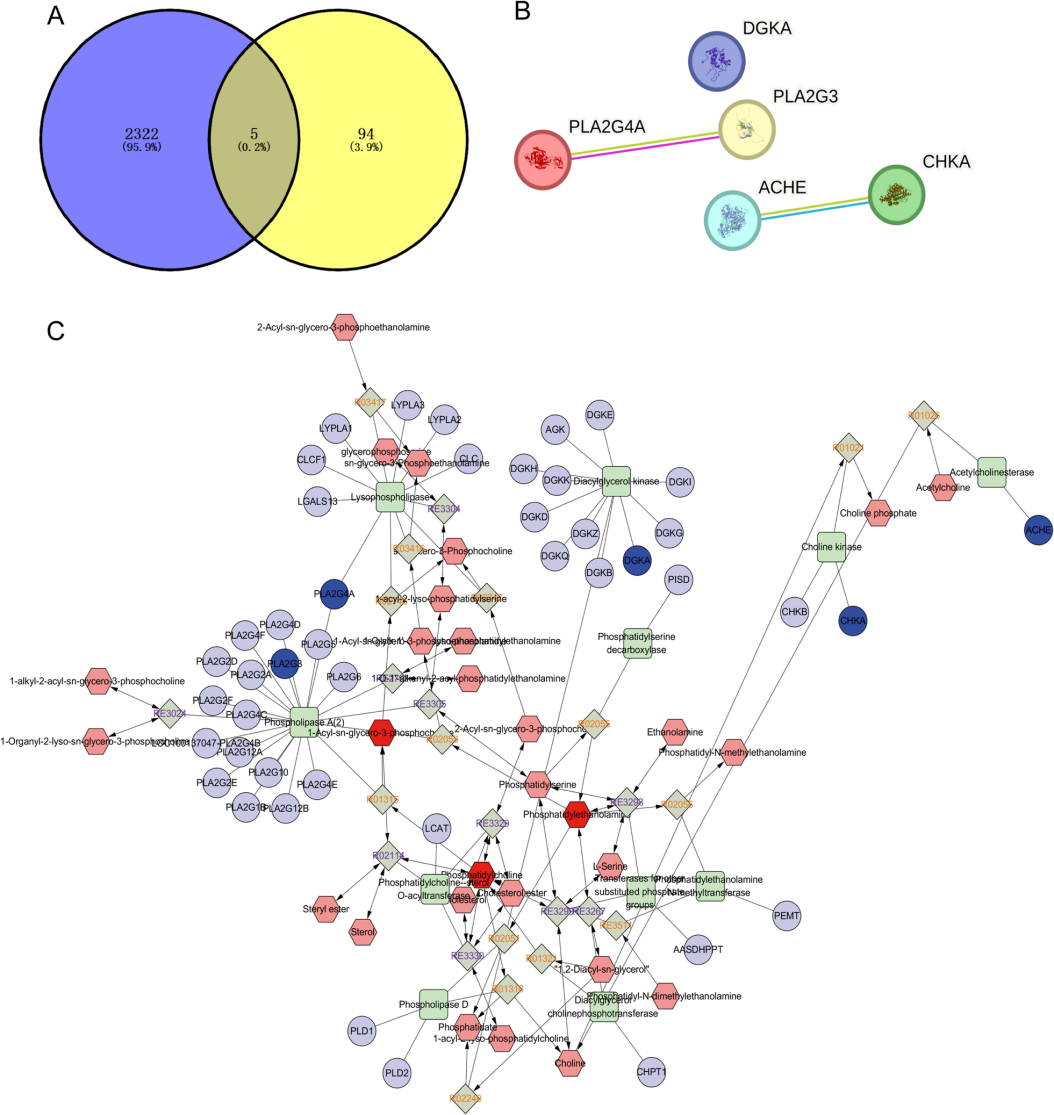
PLA2G4A, PLA2G3, DGKA, ACHE, and CHKA may be potential targets for regulating endogenous lipids in platin-resistant GC patients. **A** Distribution of Platinum resistance and Glycerophospholipid metabolism target genes. **B** Protein–protein interaction (PPI) network revealed the interaction of PLA2G4A, PLA2G3, DGKA, ACHE, and CHKA. **C** Lipid metabolite-reaction-enzyme-gene network. The red hexagons represent the detected lipids and other metabolite biomarkers in our study. Pink hexagons represent metabolites participating in the same metabolic pathway but were not detected in our study. The dark green diamonds represent the metabolic reaction of the compound. The green squares represent the enzyme. The purple circle represents genes

In this study, glycerophospholipid metabolism was the top-altered pathway in the platinum-resistant samples. To explore the target of regulating the lipid profile, we first extracted the 99 genes related to the glycerophospholipid metabolism pathway in KEGG database and then identified the gene of platinum resistance in the Genecard database with 2327 genes. As shown in Fig. 5A, five common genes were found in both pathways. DGKA, PLA2G4A, PLA2G3, ACHE and CHKA were the potential targets that regulated the biomarkers we found above via the glycerophospholipid metabolism pathway. We then explored the protein–protein interaction (PPI) network for these targets using the STRING database. As shown in Fig. 5B, PLA2G4A established interaction with PLA2G3, and likewise, ACHE interacted with CHKA. However, no interaction existed between DGKA and them. Next, network of metabolites were constructed with Metscape. We pinpointed 15 potential biomarkers via lipidomic analysis and five potential regulatory genes through target screening and bioinformatic analysis. To associate them, we employed Cytoscape to construct a lipid metabolite reaction-enzyme-gene network aiming to elucidate this process. A pathway-based network was constructed through MetScape, in which significantly altered biomarkers were linked to relevant metabolites and lipids in the same pathway. Meanwhile, all the compounds enriched in the MetScape network were annotated via the MetDisease plugin. As shown in Fig. 5C, PLA2G4A and PLA2G3 belong to the Phospholipase A (2) family, DGKA belongs to the diacylglycerol kinase family, CHKA is a choline kinase and ACHE is an acetylcholinesterase. They govern a range of metabolites via multiple metabolic pathways encompassing Arachidonic acid metabolism, Linoleic acid metabolism, and Glycerophospholipid metabolism, among others. It can be concluded that PLA2G4A, PLA2G3, DGKA, ACHE, and CHKA modulated lipid profiles in platinum-resistant GC patients through multifaceted metabolic pathways.

### PLA2G4A, DGKA and ACHE regulated lipid profiles in platinum-resistant GC patients through glycerophospholipid metabolic pathways

**Fig. 6:**
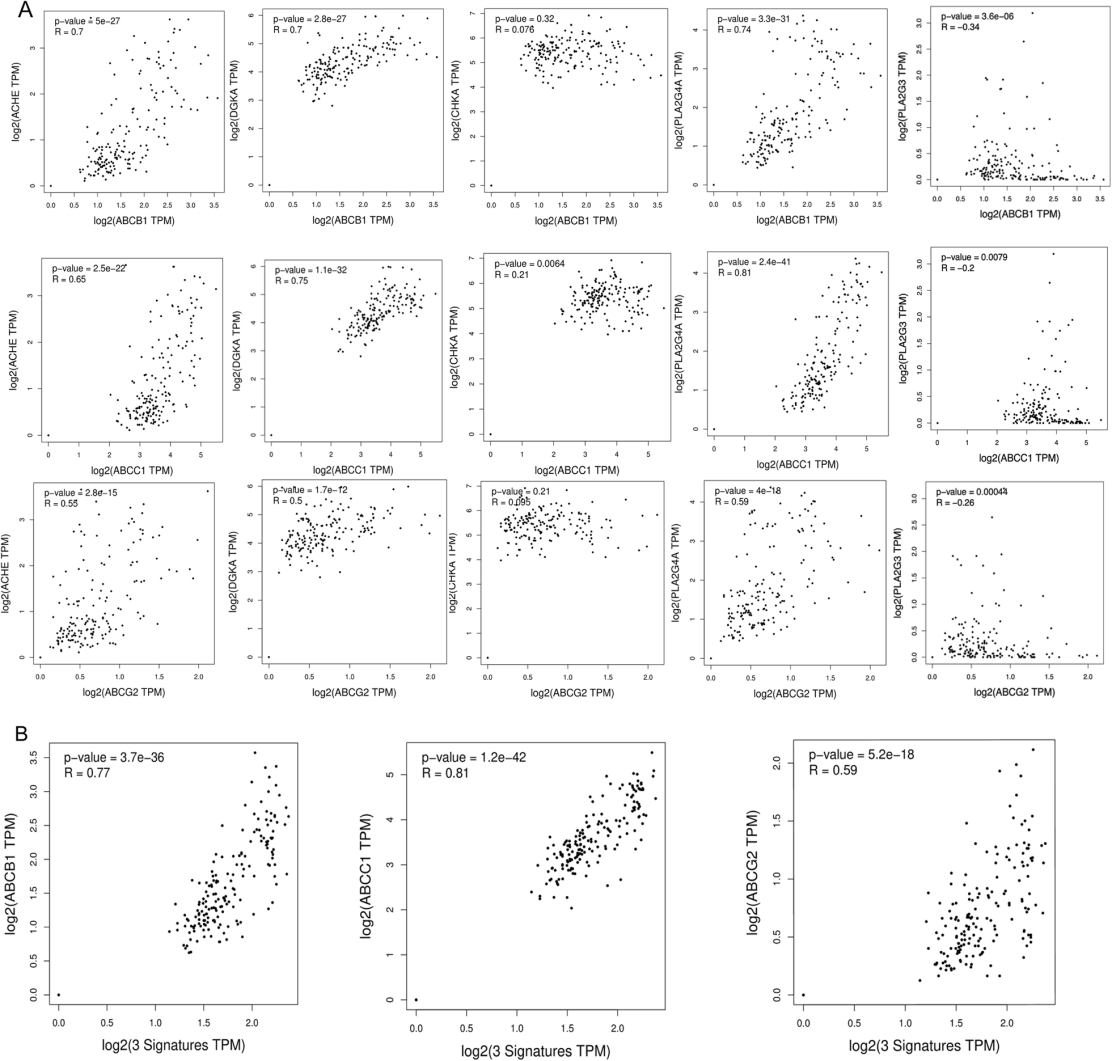
PLA2G4A, DGKA and ACHE regulated lipid profiles in platinum-resistant GC patients through glycerophospholipid metabolic pathways. **A** Utilizing the GTEx-stomach database, we conducted a Spearman correlation coefficient analysis to elucidate the interrelationships among DGKA, PLA2G4, ACHE, CHKA and the drug-resistant genes ABCB1, ABCC1, ABCG2. **B** Utilizing the GTEx-stomach database, we validated the association between the tri-gene model, comprising PLA2G4A, DGKA, and ACHE, and drug resistant genes ABCB1, ABCC1, ABCG2

Extensive research indicates that the upregulation of ATP-binding cassette (ABC) transporters is implicated in conferring resistance to chemotherapeutic agents [19]. To gain a more comprehensive understanding, ACHE, DGKA, CHKA, PLA2G4 and PLA2G3, these genes can serve as viable targets for developing drug-resistant therapy strategies. We examined the correlation analysis between the potential therapeutic targets and the ABC transporters (ABCB1, ABCC1, and ABCG2). As shown in Fig. 6A, our analysis revealed a strong positive correlation between ACHE and ABCB1, ABCC1, and ABCG2, with ABCB1 showing the highest correlation coefficient (R = 0.7). Conversely, CHKA showed weak correlations with these transporter genes. PLA2G3 exhibited a negative correlation with ABCB1, ABCC1 and ABCG2. In contrast, DGKA and PLA2G4A also exhibited notable correlations with them, with the strongest association evident with ABCC1 (R = 0.75 and R = 0.81, respectively). This underscores the potential roles of these proteins in drug resistance, underlining their significance as therapeutic targets. Finally, we evaluated the correlation of a three-gene combination model, derived from above, with each drug resistance marker individually. The generated models exhibited a high correlation coefficient with ABCB1(R = 0.77), ABCG2 (R = 0.59), and ABCC1 (R = 0.81) respectively (Fig. 6B), indicating a significant association between these gene combinations and drug resistance markers. Our results revealed that DGKA, PLA2G4 and ACHE could be used as key targets to regulate the endogenous lipids in platinum-resistant GC patients through the glycerophospholipid metabolism pathway.

### ACHE and PLA2G4A were up-regulated in platinum-resistant GC patients

**Fig.7:**
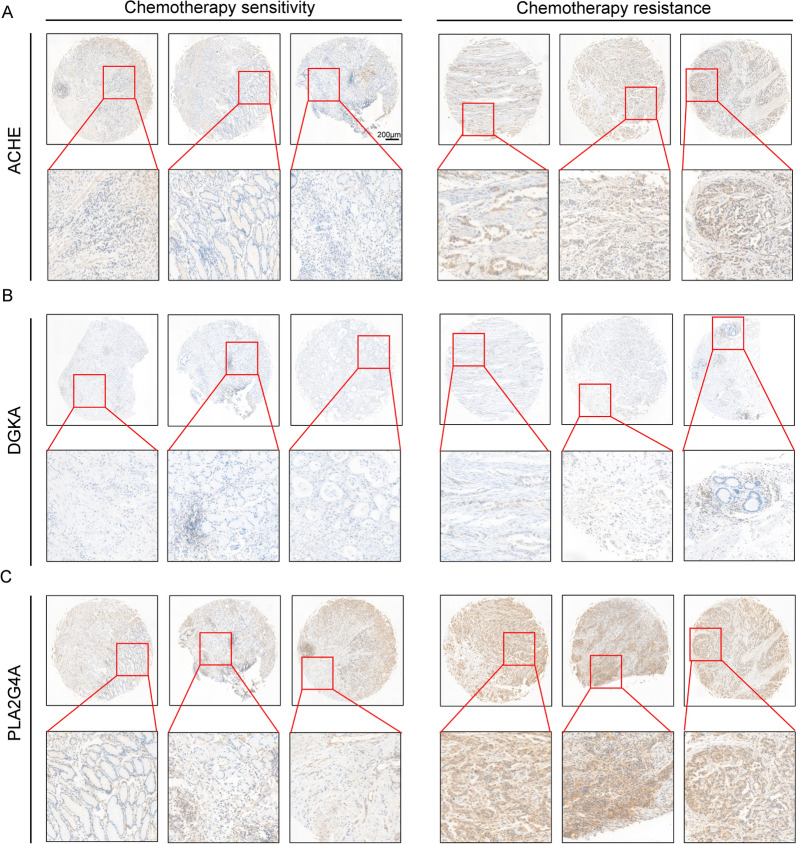
ACHE and PLA2G4A were over-expression in platinum-chemotherapy-resistant GC patients **A** Representative images of IHC staining of ACHE in GC chemotherapy sensitivity and resistance patient-tissues. **B** Representative images of IHC staining of DGKA in GC chemotherapy sensitivity and resistance patient-tissues. **C** Representative images of IHC staining of PLA2G4A in GC chemotherapy sensitivity and resistance patient-tissues. Scar bar = 200 μm

In our target screening and correlation analysis, PLA2G4A, DGKA, and ACHE emerged as potential targets to mitigate resistance in platinum-resistant GC patients, potentially achieved through the modulation of the glycerophospholipid metabolism pathway. To substantiate our results, we performed immunohistochemistry (IHC) analysis of our screened targets in a Tissue Microarray (TMA) containing a selection of platinum-based chemotherapy sensitive/resistant patients covered in this research. According to IHC from tissue microarray, we identified a notably higher expression of ACHE in the GC chemotherapy-resistant samples compared to the GC chemotherapy sensitivity tissues (Fig. 7A). DGKA was not overexpressed like PLA2G4A in GC chemotherapy-resistant samples, and only showed some elevated expression (Fig. 7B and C). Overall, our results identified the critical role of ACHE and PLA2G4A in platinum-resistant GC patients.

## Discussion

With the advent of effective tools to study lipids, including mass spectrometry-based lipidomics, lipids are emerging as central players in cancer biology [6]. Techniques such as nuclear magnetic resonance (NMR), gaschromatography-mass spectrometry (GC–MS), and liquid chromatography-tandem mass spectrometry (LC–MS) are primarily used for metabolomic analysis [20], with LC–MS-based metabolomics gaining popularity due to its extensive metabolite coverage. Recent advancements in LC and MS have further enhanced the detection sensitivity and data reliability for cancer metabolomic studies [21, 22]. In this investigation, we identified a range of lipid metabolic biomarkers linked with the prognosis of platinum resistance in GC patients. The objective was to improve the diagnosis for platinum resistance in GC patients. We screened a total of 15 metabolites as potential biomarkers and identified 2 regulatory genes (ACHE and PLA2G4A) that could potentially influence endogenous lipid profiles in platinum-resistant GC patients through the glycerophospholipid metabolism pathway.

Platinum-based therapeutics, designated as cisplatin, carboplatin and oxaliplatin, are widely employed in clinical practice due to their potent treatment efficacy and their clarified performance mechanisms [23–25]. Nevertheless, platinum-based therapeutics lead to severe adverse effect as well as causing irreversible damage to healthy tissues [26, 27]. Meanwhile, an increasing number of patients exhibits drug resistance, thereby lowering the lethality within the tumor. Although numerous studies has been conducted on molecular biomarkers, most of the identified biomarkers failed in the validation studies [28]. Therefore, it is important to explore effective biomarkers to improve the diagnosis of drug resistance in order to help in the treatment of patients, and metabolite and lipid biomarkers offer expedited success rates, promising a significant breakthrough in precision medicine. Our results were based on the reprogramming of lipid metabolism in gastric cancer, using the technology of lipidomics to reveal biomarkers and targets associated with platinum resistance in gastric cancer. We provided new ideas for the detection of platinum resistance in gastric cancer and the development of targeted drugs. In addition, it also provided direction for early diagnosis and treatment of gastric cancer, and if there was a need to find means to control biomarkers will improve the recurrence of gastric cancer. It has been reported that serum lipid metabolites such as phosphatidylethanolamine (36:2), phosphatidylcholine (32:0), and sphingomyelin (d18:0/18:1(9Z)) could serve as markers for the early diagnosis of GC [9]. Our results will be a supplement to this, which can better detect the changes in patients with gastric cancer, so as to formulate more accurate treatment plans.

Conventional methods for detecting platinum resistance in gastric cancer (GC) predominantly rely on imaging techniques and serum markers, the accuracy of which has become increasingly debated due to the clinical complexities and heterogeneous nature of GC [29, 30].These methods can be invasive, costly, and may not offer the specificity required for early detection nor real-time treatment monitoring. Unlike these traditional approaches, our study provided a non-invasive and more accurate way for the detection and monitoring of platinum resistance. Both intrinsic tumor and TME-associated lipids can sustain the therapy-resistant cancer cell phenotype [28]. Our current study aimed to unravel the complex interplay between lipid metabolism disorders and platinum resistance in GC based on lipidomics. This novel set of biomarkers, which includes MGDG (43:11)-H, Cer(d18:1/24:0) + HCOO, and others, not only reflected the intrinsic metabolic state of cancer cells but also actively contributed to the mechanisms driving drug resistance. The high predictive performance, exemplified by Cer(d18:1 + hO/24:0) + HCOO with an area under the receiver operating curve of 0.887, emphasizes the approach's potential for superior accuracy. In our study, post-treatment-based lipidomic analysis, aimed at better improving patient prognosis and paving the way for platinum-resistant precision medicine in gastric cancer.

Lipids, typically denoted as hydrophobic or amphipathic small molecules, embody both hydrophilic and lipophilic traits. Such amphipathic lipids constitute plasma membranes, enabling cells to regulate their internal biological activities and respond to fluctuations in the external milieu, such as ABC family mediated drug efflux-induced drug resistance [31]. As we all know, cellular membranes predominantly comprise three classes of lipids: (1) Glycerophospholipids (PLs), encompassing phosphatidylcholine (PC), phosphatidylserine (PS), phosphatidylethanolamine (PE), phosphatidylglycerol (PG), phosphatidylinositol (PI), and phosphatidylinositol phosphates (PIPs); (2) Sphingolipids, encapsulating sphingomyelins (SM) and glycosphingolipids; (3) Sterols, primarily cholesterol (Chol) in mammalian cells. Acting as vital constituents of biological membranes, glycerophospholipids maintain a minimum of one O-1-acyl, O-1-alkyl, or O-1-alkenyl residue affixed to the glycerol entity. Later, during glycerophospholipid metabolism, various bioactive lipid molecules, including inositol trisphosphate, diacylglycerol, arachidonic acid, phosphatidic acid, and lysophosphatidic acid, are produced, exerting regulatory influence over different cellular signaling pathways [32]. Previous studies have shown that glycerophospholipid metabolism played a significant role in proliferation of esophageal squamous cell carcinoma and breast cancer [33, 34]. However, the search for biomarkers of platinum-resistant GC and the discovery of related regulatory genes are still inadequate. Our study not only discerned that the differential lipids between the chemo-sensitive and chemo-resistant cohorts were strongly enriched within the glycerophospholipid metabolism pathway but also innovatively proposed targets against glycerophospholipid metabolism.

In recent years, targeted therapies for cancer have achieved remarkable results [35]. Determining targetable gene and biomarkers as alternative or combinational treatments can add to the clinical efficacy of the current therapies and overcome potential resistance [36]. In our study, DGKA, CHKA, PLA2G3, PLA2G4A, and ACHE have been preliminarily identified as potential targets for overcoming platinum-chemotherapy-resistant GC through glycerophospholipid metabolism. Diacylglycerol kinase α (DGKA), the inaugural member of the DGK family, is known for its phosphorylation of diacylglycerol (DAG) into phosphatidic acid (PA), modulating lipid metabolism [37]. Evidence suggested that DGKA and its product PA may function as mediators of platinum resistance in ovarian cancer [38]. Analogously, our research suggested that DGKA might offer a new therapeutic target for mitigating platinum resistance in gastric cancer. Choline kinase alpha (CHKA) is integral to the Kennedy pathway, phosphorylating free choline (Cho) into PC. Upregulation of CHKA in multiple cancers, including gastric cancer, majorly contributes to augmented PC levels [39–42]. Inhibiting CHKA could potentially amplify platinum-based chemotherapy sensitivity in ovarian cancer [43]. Both PLA2G3 and PLA2G4A are members of the Phospholipase A2 family (PLA2s), which hydrolyze the sn-2 acyl bond of glycerophospholipids (GPLs) to yield lysophospholipids (LPLs) and free fatty acids [44], playing pivotal roles in carcinogenesis. Elevated expression of Group III Phospholipase A2 (PLA2G3) in human colorectal adenocarcinoma tissues has labeled it as a diagnostic biomarker of colorectal carcinoma [45]. Moreover, high levels of group IVA cPLA2 (PLA2G4A) have been associated with a dismal prognosis in perihilar cholangiocarcinoma (PHCCA) and distal cholangiocarcinoma [46]. In our study, we emphasized PLA2G4A and ACHE were the key targets for overcome platinum resistance in GC. Some evidence points towards high PLA2G4A expression in gastric cancer, and inhibiting PLA2G4A has been proposed as a therapeutic strategy to counteract chemo-resistance in this malignancy [16, 47]. However, targeting PLA2G4A as key evidence against drug resistance is still lacking. Acetylcholinesterase (ACHE), a crucial enzyme in catalyzing the hydrolysis of cholinergic neurotransmitters, has been linked to various roles in cancer pathogenesis [48, 49]. Inhibition of ACHE reportedly promotes cell death [50]. However, it has only been reported to be associated with resistance in ovarian cancer [51], and there is no evidence of platinum resistance in GC. Together, our study innovatively revealed PLA2G4A and ACHE as targets of platinum chemotherapy resistance in GC from a clinical perspective based on lipidomics.

While we have identified PLA2G4A and ACHE as notable targets and a variety of lipid-biomarker in platinum-resistant GC, we acknowledged the limitations stemming from LC/MS- mediated lipidomics as a singular analytical technique, the confined sample size, and the limited scope of the study population. We fully recognized that our research represents only a fraction of the exhaustive work required to translate these biomarkers into clinically viable tools. Enhancing the clinical relevance of our findings should base on larger sample sizes that include racial and regional differences, etc. Moreover, the precision of biomarker identification can be significantly enhanced through the application like targeted lipidomics. Meanwhile, a more holistic exploration of the full biological spectrum could be processed to enrich our understanding of platinum-resistant GC by proteomics and genomics.

## Conclusion

In summary, we identified 15 lipid metabolites through lipid metabolomics as biomarkers for diagnosing the platinum resistance in gastric cancer patients, which greatly improve for diagnosis of platinum-based chemotherapy resistance in gastric cancer patients. Moreover, we identified 2 potential targets (PLA2G4 and ACHE) for combating platinum chemotherapy resistance in gastric cancer treatment though glycerophospholipid metabolism. These results not only strength our understand of mechanism underlying platinum-based chemotherapy resistance but also provide a new insight about the development of target drug.

## Data Availability

All data generated or analyzed during this study are included in this published article.

## Abbreviations

ACHE: Acetylcholinesterase
CR: Chemotherapy resistance
CS: Chemotherapy sensitivity
CHKA: Choline kinase alpha
DGKA: Diacylglycerol kinase α
FC: Foldchange
GC: Gastric cancer
IHC: Immunohistochemical
KEGG: Kyoto Encyclopedia of Genes and Genomes
LC–MS: Liquid Chromatograph Mass Spectrometer
MDR: Multidrug resistance
PC: Phosphatidylcholines
PCA: Principal component analysis
PE: Phosphatidylethanolamine
PLA2G3: Group III Phospholipase A2
PLA2G4A: Group IVA Phospholipase A2
PLS-DA: Partial least squares discriminant analysis
PPI: Protein–protein interaction
QC: Quality control
ROC: Receiver Operating Characteristic
SM: Sphingomyelin
VIP: Variable importance in projection
